# Family Value Matters: Intergenerational Solidarity and Life Satisfaction of Chinese Older Migrants

**DOI:** 10.1093/geroni/igaa057.1109

**Published:** 2020-12-16

**Authors:** Jun Yao, Li Zhang, Peiyi Lu

**Affiliations:** 1 NANJING MEDICAL UNIVERSITY, NANJING, Jiangsu, China; 2 Nanjing University of Chinese Medicine, NANJING, Jiangsu, China; 3 Iowa State University, Ames, Iowa, United States

## Abstract

The number of older adults who migrate due to family reasons has been increasing rapidly in China in the past decade. However, few empirical studies have focused on this group. This study focused on them and explored the association of intergenerational solidarity with older migrants’ life satisfaction when they were adapting to the new environment. We surveyed 340 older adults who migrated to Nanjing with their children either to help care for the grandchildren or enjoy retirement life. Respondents were recruited from the community. Structural equation modeling was adopted to analyze the associations among intergenerational solidarity, loneliness, aging perception, and life satisfaction. Results showed intergenerational solidarity was negatively correlated with loneliness (r=-0.304) but positively correlated with life satisfaction (r=0.386). Loneliness was linked to lower life satisfaction(r=-0.517). Path analyses showed that loneliness played a partial mediation role on the relationship of intergenerational solidarity and life satisfaction. Aging perception negatively moderated the association between intergenerational solidarity and loneliness, and also negatively moderated the mediating effect of loneliness on intergenerational solidarity and life satisfaction. It is concluded family values played important roles in Chinese older migrants’ mental health. When they migrate to a new city, intergenerational solidarity can help ease their loneliness and subsequently improve their life satisfaction, which finally help them adapt to the new environment. Positive perception towards aging also helps improve their well-being after migration. Based on these findings, we suggest practitioners design education program to promote family values among the family with older migrants.

